# Mobility and HIV care engagement: a research agenda

**DOI:** 10.1002/jia2.26058

**Published:** 2023-03-21

**Authors:** Marguerite Thorp, James Ayieko, Risa M. Hoffman, Kelvin Balakasi, Carol S. Camlin, Kathryn Dovel

**Affiliations:** ^1^ Division of Infectious Diseases David Geffen School of Medicine University of California Los Angeles Los Angeles California USA; ^2^ Center for Microbiology Research Kenya Medical Research Institute Kisumu Kenya; ^3^ Partners in Hope Lilongwe Malawi; ^4^ Department of Obstetrics Gynecology & Reproductive Sciences University of California San Francisco San Francisco California USA; ^5^ Center for AIDS Prevention Studies Department of Medicine University of California San Francisco San Francisco California USA

**Keywords:** HIV, mobility, migration, sub‐Saharan Africa, antiretroviral therapy, outcomes

## Abstract

**Introduction:**

Mobility is common and an essential livelihood strategy in sub‐Saharan Africa (SSA). Mobile people suffer worse outcomes at every stage of the HIV care cascade compared to non‐mobile populations. Definitions of mobility vary widely, and research on the role of temporary mobility (as opposed to permanent migration) in HIV treatment outcomes is often lacking. In this article, we review the current landscape of mobility and HIV care research to identify what is already known, gaps in the literature, and recommendations for future research.

**Discussion:**

Mobility in SSA is closely linked to income generation, though caregiving, climate change and violence also contribute to the need to move. Mobility is likely to increase in the coming decades, both due to permanent migration and increased temporary mobility, which is likely much more common. We outline three central questions regarding mobility and HIV treatment outcomes in SSA. First, it is unclear what aspects of mobility matter most for HIV care outcomes and if high‐risk mobility can be identified or predicted, which is necessary to facilitate targeted interventions for mobile populations. Second, it is unclear what groups are most vulnerable to mobility‐associated treatment interruption and other adverse outcomes. And third, it is unclear what interventions can improve HIV treatment outcomes for mobile populations.

**Conclusions:**

Mobility is essential for people living with HIV in SSA. HIV treatment programmes and broader health systems must understand and adapt to human mobility, both to promote the rights and welfare of mobile people and to end the HIV pandemic.

## 1 INTRODUCTION

In the fight to end the HIV pandemic, it has become more important than ever to focus on marginalized groups that struggle to remain in care. The world is nearing UNAIDS’ 95‐95‐95 targets—ensuring 95% of people with HIV know their status, 95% of those who know their status are engaged in treatment and 95% of those in care have achieved viral suppression [1]—but progress has been slower for certain groups, including mobile people. Mobility threatens each stage of the care cascade, especially in sub‐Saharan Africa (SSA): mobile people are less likely to access preventative services, less likely to remain on antiretroviral therapy (ART), and less likely to achieve viral suppression as compared to non‐mobile people [2, 3, 4, 5, 6]. These disparities are a problem both for mobile people who experience worse HIV‐related outcomes, and for larger goals of epidemic control since mobility is simultaneously associated with viremia, higher rates of multiple and concurrent partners [7], and increased risk of HIV transmission [3, 8]. Ending the HIV pandemic requires understanding the challenges faced by mobile populations and designing effective systems to address them.

Mobility is an essential livelihood tactic for those in SSA. People in SSA use mobility to find employment, to stay connected with family and to avoid conflict or disasters [9, 10, 11, 12]. People face similar pressures regardless of HIV status, and in some cases, people with HIV may experience higher levels of mobility due to HIV‐related factors, such as marital dissolution or stigma [13, 14, 15]. Yet, most HIV treatment programmes do not accommodate mobile clients [16].

Defining “mobility” is contentious, and researchers use widely varying measures and definitions [16], ranging from one night away in the past 6 months to 30 nights away in the past year. It may be appropriate to use different definitions of mobility in different settings, since the impact of mobility on the use of health services is likely context‐dependent [2]. However, widely varying definitions make it difficult to achieve a cohesive understanding of the relationship between mobility and HIV care [17].

We review the current landscape of mobility and HIV care research to identify what is already known, gaps in the literature, and recommendations for future research. Of note, many mobility‐related challenges apply not only to HIV, but also to any chronic disease, such as hypertension, diabetes, and other conditions that require repeated, regular engagement with the health system. HIV provides a useful lens to examine chronic disease care and access issues for mobile people due to available data and well‐established delivery systems. HIV also presents unique issues of stigma and disclosure, which may further affect adherence for mobile people.

## 2 DISCUSSION

### 2.1 Sources of mobility

Mobility is common in SSA and is expected to increase in the coming decade [9]. Most movements within the region are temporary and recurrent. Informal employment is the norm [18], and jobs are often short‐term and insecure—however, because cash‐earning opportunities are inconsistently available in rural areas, such “piece work” remains highly desirable even if it requires movement. Within countries, modern cities offer greater earning opportunities, which may induce permanent or temporary movement; however, a variety of factors (including the high costs of urban living, familial ties and social norms) may pressure low‐wage workers to maintain rural residences [19, 20, 21]. Rural–rural migration is increasingly recognized in mobility research as well: seasonal patterns present economic opportunities in different parts of the country depending on the time of the year, and geographically dispersed social networks result in travel for caregiving, funerals and celebrations [10]. Between countries, the demand for labour and workers’ desire for higher wages attract workers from lower‐income countries to more industrialized neighbours, though immigration laws and social pressures may cause them to eventually return [22].

Increasing civil conflict, violent extremism, and rising authoritarianism have led to record numbers of displaced people in the 21st century [11, 23], and a changing climate has caused disasters and made some environments uninhabitable [12, 24]. Extreme climate or conflict events can result in a mix of permanent and temporary mobility. Individuals may flee and later return home as climate challenges wane or as levels of violence fluctuate. Workers may move seasonally to newly productive regions and away from barren ones [25, 26]. These patterns will continue, and with regard to climate change, movements are likely to increase over time [12, 27].

Studies on mobility tend to focus on *migration* (a permanent or semi‐permanent change of residence) because census data describe who relocates where and when [7, 15, 28]. Temporary mobility is more difficult to study—including circular movements between multiple residences, temporary relocation for better earning opportunities, or frequent travel for trading goods. Estimates of the prevalence of temporary mobility are highly variable, and traditional models for estimating mobility that rely only on distance and population density may not be applicable in SSA [29]. Several studies using cell phone data demonstrate how frequent mobility may be: in Rwanda, 32% of cell phone owners spent 3–12 months outside their home district in the 4 years between 2005 and 2008 [30]; in Kenya and Namibia, 5% and 13% of cell phone users travelled across district borders on any given day, respectively [31]. Temporary mobility may have similar or greater impacts on HIV care outcomes as compared to permanent migration. For example, a single permanent move may transiently interrupt access to HIV care, but with time individuals may be able to integrate and re‐start treatment [32]. Temporary mobility, on the other hand, presents a frequent risk for missed appointments and treatment interruptions that can negatively impact long‐term outcomes [33, 34].

### 2.2 What is known about mobility and HIV care engagement

Early epidemiologic research on HIV showed associations between mobility and HIV acquisition, suggesting that highly mobile individuals have a higher risk of HIV infection [7, 35, 36, 37]. More recent data explore the role of mobility in HIV treatment outcomes. Though quantitative data on rates of HIV testing among mobile people are scant [38], multiple studies show that mobility is negatively associated with retention in HIV care. In high‐mobility settings like the Kenya–Uganda border, rural Lesotho, and peri‐urban areas of South Africa, mobility is clearly associated with poor HIV treatment outcomes [2]: mobile people are less likely to be retained in treatment [4, 39, 40] and less likely to be virally suppressed [4, 5, 6]. The magnitude of mobility's effects ranges widely—in part because definitions of mobility are so varied—from a 47% reduction in viral suppression to a six‐fold reduction in 1‐year retention. Mobile people are also less likely to be retained in pre‐exposure prophylaxis (PrEP) programmes [41, 42]. Several qualitative studies from diverse settings, including West, East and Southern Africa, demonstrate how mobility can trigger a chain of events leading to treatment interruption [13, 43, 44, 45, 46, 47]. A single missed appointment can have significant consequences: it may result in long periods of treatment interruption due to the logistical challenges of re‐establishing care and fear of negative patient–provider interactions (i.e. being punished for being a “defaulter”) [48, 49]. Additionally, treatment interruption can diminish the internal connection patients feel towards HIV care [43]. Those who experience treatment interruption are more likely to have repeat interruptions over time and stop ART completely [50, 51, 52].

### 2.3 Closing the gaps in understanding

While poorer outcomes for mobile people are clearly documented, there is much less known about the mechanisms through which mobility affects HIV outcomes and what to do about it. Pressing questions include: What aspects of mobility cause the greatest threat to ART retention (i.e. what is “high‐risk” mobility)? What populations experience high‐risk mobility? And what interventions can improve ART outcomes for mobile people?

#### 2.3.1 What aspects of mobility matter?

We must better understand which aspects of mobility—including destinations, temporality (including duration, frequency, or seasonality), purpose and level of an individual's control over travel—impede ART engagement and if there are high‐risk mobility characteristics that interventions should target [17, 34]. Not all mobility is the same, and various forms of mobility will affect HIV care differently. Specific patterns of mobility vary by region and individual, and an individual's experience may vary over time [53].

Members of our team have found that duration and individual agency over trip departure and duration matter. We recently assessed the impact of mobility on ART adherence (measured by levels of ART in hair samples) in communities in the Lake Victoria region and found that longer trips (>1 month) were associated with lower ART adherence as compared to shorter trips [54]. We also conducted qualitative research with mobile men in Malawi and found that men's control (or agency) over travel departure and duration greatly impacted how mobility interacted with HIV care [55]. Men emphasized that unplanned travel was unavoidable due to limited cash‐earning opportunities at home; they had no choice but to travel wherever employment was available and for whatever duration an employer demanded. This meant that men often could not return to their assigned ART clinic before or during travel, resulting in treatment interruption. There may be other key aspects that characterize higher‐ versus lower‐risk mobility for ART retention, although there is very little research on the topic.

Future research must explore what aspects of mobility matter for HIV care, rather than treating mobility as a singular phenomenon leading to a uniform risk of treatment interruption. Definitions of “high‐risk mobility” may be context‐ and population‐dependent. Research may require detailed qualitative studies or novel techniques for analyzing survey data (such as the visual analysis used in a study of mobility among female sex workers in Zimbabwe [56]) before locally specific definitions of high‐risk can be operationalized. Whether there are universal aspects of mobility that matter for ART outcomes in all settings is not yet clear, but may emerge from a synthesis of more localized studies.

#### 2.3.2 Who is at risk?

Just as mobility patterns vary, so too do mobile clients and their ability to navigate mobility and remain in care [17]. Key questions to explore include whether certain populations are at increased risk of treatment interruption due to mobility—or, alternatively, whether there are specific populations that can overcome mobility‐related barriers to care more easily. Answering these questions will help tailor interventions to those who need it most. For example, should interventions target people in extreme poverty who have limited employment opportunities, or those with more education who seek better job prospects in cities [57, 58]? Many men in SSA depend on mobility, but men who experience relative and absolute poverty often participate in more chaotic, less predictable mobility in pursuit of work [55], and disaggregating this broad group of “mobile men” could help target future interventions.

We know that women in SSA are also highly mobile, though their forms of mobility tend to differ from men's—women's movements have been shorter‐term and more localized, with more frequent returns to households of origin [45, 59]. How do these different patterns of mobility intersect with gendered patterns of HIV care outcomes? Relatedly, many studies find high rates of mobility among female sex workers, especially in West and Central Africa, but whether mobility is a risk factor or survival strategy for this vulnerable population is less clear [56, 60, 61, 62, 63]. Children and adolescents may move with their caregivers, and they may face additional mobility‐related challenges if attending boarding school [64, 65]; this vulnerable population faces many treatment adherence challenges already, which may be compounded by mobility [66, 67]. Finally, men who have sex with men (MSM) may experience high rates of mobility and unique challenges for engaging in care, and the few studies available suggest that mobility is associated with worse PrEP and ART outcomes among MSM. This population is generally understudied in SSA [68, 69].

Research should also examine if there are other key factors that amplify the negative impacts of mobility, such as disclosure, social support (at the destination and at home), literacy, and economic status [28]. It might be possible to use events like the COVID‐19 pandemic or climate disasters to understand how mobility interacts with other sources of vulnerability. For example, the COVID‐19 pandemic—which impeded mobility for some populations but created a new need for mobility among others [70, 71]—may provide interesting research opportunities to understand whether mobility itself causes poor ART outcomes, or if it is a marker for other types of vulnerability that are associated with poor outcomes. Finally, how do climate change, seasonal trends [72, 73], and fluctuating levels of violent conflict affect mobility decisions [74]? The ability to predict individuals’ mobility and its impact on HIV outcomes, rather than just respond to it reactively, will be essential to target health system interventions.

#### 2.3.3 Exploring solutions

Further research is needed on interventions to improve ART outcomes for mobile populations. Client‐centred, differentiated service delivery (DSD) models [75] should work for mobile populations. To date, DSD options have been largely available only to stable clients retained in care for more than 6 months, but there is increasing momentum towards expanding DSD to new or high‐risk clients [75, 76]. Given the barriers mobile people face related to frequent facility visits for ART refills, they may benefit substantially from DSD models that reduce the burden of care.

Several DSD models could be considered for mobile populations. First, multi‐month dispensing (MMD) for up to 6 months of ART is now common practice for stable clients and is highly acceptable, feasible, and effective from client and health system perspectives [77, 78, 79]. The shift to MMD was accelerated by the COVID‐19 pandemic to reduce the need for people to make frequent return visits to health facilities and may partly explain the unexpectedly good ART outcomes seen widely in 2020 and 2021. MMD for mobile clients would allow clients to travel for longer periods of time without running out of medications, and it would reduce the opportunities for missed appointments. Second, community‐based ART distribution has improved initiation, retention and viral suppression [80, 81, 82, 83] and may be a useful solution for highly mobile people if refills were made available in common destinations or transit hubs.

Peer mentors provide tangible, real‐life solutions to HIV care challenges, and can improve ART engagement among pregnant and breastfeeding women [84, 85], adolescents [86, 87, 88], men [89], and general populations [90, 91]. Deploying peer mentors who have experience with mobility may improve outcomes for mobile clients since this population often does not know how to navigate mobility and HIV care and the topic is rarely discussed in routine counselling sessions. In Kenya, social networks are being used to distribute HIV self‐test kits and provide ART and PrEP linkage support: highly socially connected men are trained and supported to encourage other men in their network to use HIV services, with results pending [92]. A similar approach could be used for highly mobile populations.

Long‐acting injectable therapies could help mobile clients avoid unwanted disclosure, a fear that is often magnified while travelling. While current regimens require injections every 2 months [93], the promise of injectable regimens delivered every 6 months or the use of an ART implant could substantially benefit many care recipients [94, 95].

National electronic health records would allow clients to easily access care from any facility in a given country [2, 40, 46]. Even if clients have reduced facility visits due to MMD or long‐acting ART, unplanned trips may still interfere with scheduled appointment times, and accessing care outside of one's home facility is often difficult. In Malawi, we found that mobile men often tried to access HIV care at another facility while travelling, but facility staff sometimes refused services, asking for an official transfer letter from the home facility. National electronic records would promote continuity of care, regardless of client location. If national electronic health records are not feasible, national programmes could consider communication systems that allow health workers across facilities to communicate about clients who move between facilities.

Finally, mobile phone interventions may help mobile clients find nearby facilities or leverage telemedicine to connect with their home facility [96, 97].

## 3 CONCLUSIONS

Mobility has a profound impact on HIV engagement and will likely increase in upcoming years due to climate change, conflict and unequal economic development. HIV programmes must understand what type of mobility has the greatest impact on HIV treatment outcomes, which clients experience high‐risk mobility, and how to provide high‐quality care for these clients. Temporary mobility may be much more common than originally assumed and deserves particular attention. As countries near UNAIDS’ 95/95/95 goals, finding innovative strategies to serve mobile populations is critical, both to promote the rights and welfare of mobile people themselves and to end the HIV pandemic.

## COMPETING INTERESTS

The authors have no competing interests to declare.

## AUTHORS’ CONTRIBUTIONS

MT and KD developed the concept and outline for this commentary. The first draft of the manuscript was written by MT, RMH and KD. All authors (MT, JA, RMH, KB, CSC and KD) participated in framing discussions, provided comments and edits on the initial draft and approved the final manuscript.

## FUNDING

Individual support during the preparation of this commentary included funding from the Fogarty International Center (K01‐TW011484‐01; UCLA CFAR grant AI028697; the University of California Global Health Institute D43TW009343), the Bill & Melinda Gates Foundation (grant #001423) and the National Institutes of Mental Health (T32MH080634).

## DISCLAIMER

The funders of the study had no role in study design, data collection, data analysis, data interpretation or writing of the report.

## Data Availability

This manuscript does not use original data.
